# Complication Prevention in Combined Abdominoplasty and Gynecologic Procedures: A Multidisciplinary Narrative Review

**DOI:** 10.1093/asjof/ojag193

**Published:** 2026-10-01

**Authors:** Tony Kazzi, Sergio Sbeih, Tia Sarkis, Marylyn Ayoub, Antoine Abi Abboud

## Abstract

Combined abdominoplasty (ABP) and open gynecologic surgery is an established approach that may reduce anesthesia exposure, shorten convalescence, and lower costs relative to staged procedures, yet its safety profile and optimal perioperative management remain incompletely defined, with a heterogeneous evidence base and no updated synthesis since 2007. The authors conducted a narrative review, searching PubMed, MEDLINE, and Scopus from inception to March 2026 for studies of combined open ABP and gynecologic procedures in adults; 14 studies published between 1983 and 2022 were included, comprising retrospective cohorts, case series, case–control, and observational studies, 1 literature review, and 2 case reports (sample sizes 1-269). Reported outcomes were heterogeneous: some early series described longer operative times and greater morbidity, whereas recent comparative data found combined ABP–hysterectomy associated with a complication rate comparable to or lower than staged surgery (7.7% vs 12.5%; *P* = .04) alongside reduced operative and anesthesia times. Key modifiable risk factors include smoking, obesity, and the American Society of Anesthesiologists class. Formal venous thromboembolism risk stratification using the Caprini model with risk-adjusted prophylaxis, prophylactic antibiotics, maintenance of normothermia, separate instrument sets, and coordinated multidisciplinary planning are the principal evidence-based strategies for complication prevention. Rare delayed complications, notably abdominal-wall endometrioma, are consistently missed by short-term follow-up. Combined ABP and open gynecologic surgery can be performed safely in appropriately selected patients, although the evidence is limited by retrospective designs and outcome heterogeneity; prospective multicenter studies with standardized reporting and long-term follow-up are needed.

**Level of Evidence:** 3 (Therapeutic) 
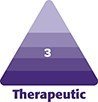

Over the past several decades, interest in combining aesthetic abdominal contouring with open gynecologic surgery has grown considerably, reflecting a broader shift toward comprehensive surgical management within a single operative episode. Patients undergoing procedures such as abdominal hysterectomy increasingly request concurrent abdominoplasty (ABP) to address both functional and aesthetic concerns simultaneously.^1,2^ Advances in surgical technique and perioperative care have made such combined approaches technically feasible and clinically attractive.

The rationale for this combination is multifaceted. Performing ABP concurrently with a gynecologic procedure reduces total anesthesia exposure, cumulative operative risk, and overall recovery time relative to 2 staged operations, while also lowering costs through reduced perioperative resource duplication and shorter hospitalization.^3,4^ However, several studies have demonstrated that combined procedures are associated with increased operative time, greater intraoperative blood loss, and a higher incidence of complications—most notably venous thromboembolism (VTE)—particularly in high-risk patient populations.^1^ More recent large-scale analyses suggest that, with appropriate patient selection and meticulous perioperative planning, the combined approach can be performed safely without a statistically significant increase in overall complication rates.^5,6^

Despite growing clinical interest, the evidence base remains limited in quality and heterogeneous in design. The existing literature consists predominantly of small retrospective case series, case–control studies, and isolated case reports, with few standardized outcome measures and limited long-term follow-up.^3,4,7^ To date, only one narrative review specifically addressing this topic has been published.^8^ Given the subsequent emergence of large database analyses and evolving perioperative protocols, an updated synthesis is warranted.

A multidisciplinary approach integrating plastic and gynecologic surgical expertise is central to optimizing patient selection, operative planning, and postoperative outcomes. Accordingly, the primary aim of this narrative review is to provide an updated, critical appraisal of the combined ABP and open gynecologic procedure literature, with a focus on complication prevention and multidisciplinary management strategies.

## METHODS

### Search Strategy

A narrative review of the literature was conducted in accordance with standard methodology for narrative reviews. Three electronic databases were searched: PubMed, MEDLINE, and Scopus. The search was performed from database inception to March 2026, with no language restrictions applied at the search stage. The database search was performed independently by 2 authors (M.A. and T.S.). Retrieved records were screened against the eligibility criteria independently by 2 further authors (T.K. and S.S.), and disagreements regarding study inclusion were resolved by consensus discussion, with the senior author (A.A.A.) adjudicating when consensus could not be reached.

For PubMed and MEDLINE, the following search string was used: (“abdominoplasty” OR “tummy tuck”) AND (“gynecologic surgery” OR “hysterectomy” OR “oophorectomy” OR “myomectomy”) AND (“complications” OR “safety” OR “perioperative management” OR “risk reduction”). For Scopus, the equivalent title–abstract–keyword field search was applied: TITLE-ABS-KEY(“abdominoplasty” OR “tummy tuck”) AND TITLE-ABS-KEY(“gynecologic surgery” OR “hysterectomy” OR “oophorectomy” OR “myomectomy”) AND TITLE-ABS-KEY(“complications” OR “safety” OR “perioperative management” OR “risk reduction”). Reference lists of all identified relevant articles were hand-searched to identify additional studies not captured by the electronic search.

### Inclusion and Exclusion Criteria

Studies were included, if they: (1) reported on adult patients (age ≥18 years) undergoing combined open ABP and gynecologic surgery; (2) provided data on perioperative outcomes, complications, or management strategies; and (3) were published in peer-reviewed journals. All study designs were eligible for inclusion, including randomized controlled trials, prospective and retrospective cohort studies, case–control studies, observational studies, case series, case reports, and narrative or systematic reviews.

Studies were excluded, if they: (1) reported exclusively on minimally invasive gynecologic procedures performed without an open abdominal component; (2) described ABP combined with nongynecologic procedures only; (3) were published in languages other than English without an available translation; or (4) were conference abstracts, editorials, or letters without original data. Duplicate publications reporting on the same patient cohort were excluded, retaining the most recent or comprehensive report. Minimally invasive gynecologic procedures performed without an open abdominal component were excluded because the present review specifically concerns the interaction between ABP and a shared open abdominal operative field; laparoscopic and transvaginal approaches differ fundamentally in access, insufflation, and abdominal-wall disruption, and combining them with ABP raises distinct considerations (eg, the effect of pneumoperitoneum on flap perfusion and the absence of a shared laparotomy incision) that warrant separate, dedicated evaluation rather than pooled analysis with open procedures.

### Data Extraction

Data were extracted independently by 2 authors (T.K. and S.S.) using a standardized extraction form. The following variables were recorded for each included study: study design, sample size, patient demographics, gynecologic procedure performed, prophylaxis and preoperative planning measures, operative time, intraoperative blood loss and transfusion requirements, surgical techniques employed, major complications, minor complications, hospital length of stay, and study conclusions. Extracted data were synthesized narratively; formal meta-analysis was not performed owing to the heterogeneity of study designs and outcome definitions across included studies.

## RESULTS

The search strategy identified 14 studies meeting the inclusion criteria, published between 1983 and 2022 (Table 1). Study designs included retrospective cohort studies, case series, case–control studies, observational study, literature review, and case reports. Sample sizes ranged from a single patient to 269 patients in the largest cohort. The combined procedures reported across the included studies consisted predominantly of ABP performed concurrently with abdominal hysterectomy, with or without bilateral salpingo-oophorectomy (BSO); a minority of studies also included tubal ligation, myomectomy, or hernia repair as adjunct procedures. Key outcomes reported included operative time, intraoperative blood loss and transfusion requirements, major and minor complication rates, hospital length of stay, and VTE prophylaxis practices. Two case reports described delayed complications in the form of abdominal-wall endometrioma arising at the operative scar following combined ABP and hysterectomy. A full summary of included studies is presented in Table 1. The quantitative findings extracted from the included studies are summarized below and in Table 1 and are referred to in the Discussion section.

**Table 1.** Summary of Included Studies on Combined Abdominoplasty and Open Gynecologic Procedures

Study	Design	*n*	Prophylaxis/planning	Operative time	Blood loss and transfusion	Techniques	Major complications	Minor complications	Hospital stay	Conclusion
Freedom (1983)^4^	Case series	129	N/A	N/A	N/A	Langer's line incisions; subcuticular sutures; meticulous hemostasis; drainage and antibiotics	N/A	3% overall (unspecified)	N/A	Combined procedures advocated; low complication rate questioned by subsequent authors
Gemperli et al (1992)^2^	Case series	103	Aggressive DVT prophylaxis attributed to zero PE rate	Increased by 40-90 min for gynecologic component	3 patients autologous transfusion (2.9%); no allogeneic transfusions	Standard or limited ABP ± liposuction in delimited areas	None (0 PE, 0 major infections, 0 deaths)	2 minor wound complications (1.9%)	N/A	Safe when performed by experienced teams with aggressive DVT prophylaxis
Gerow et al (1996)^9^	Case series	Unspecified	Bowel rest; NGT intraoperative; nitrous oxide avoided; COPD contraindicated	+40-90 min for gynecologic component	1 significant complication in series; none related to liposuction	French-line bikini-line incisions; aggressive fascial plication; limited lateral undermining; ABP flap raised before peritoneal entry; gynecologic incisions at or near midline	1 skin slough (lower abdominal wall)—debridement, grafting, revision (nitrous oxide; since abandoned)	Suture abscess/granuloma; minor wound dehiscence; seroma	Mean 3 days	Safe, effective, and reliable; COPD is only absolute contraindication; nitrous oxide must be avoided
Kaplan and Bar-Meir (2005)^7^	Case series	15	Prophylactic antibiotics at induction; miniheparin/enoxaparin for overweight; joint preoperative planning	3.5 h (combined) vs 2.5 h ABP alone; 1.5 h TAH-BSO alone	No transfusions required	ABP incisions marked first; gynecologist incises along linea alba; PDS 2/0 closure; 2-layer rectus plication; “fox” umbilicoplasty	1 major wound infection—1-week hospitalization, drainage, IV antibiotics (recent smoker, postbariatric, 4200 g resection)	2 minor wound infections; 1 seroma (outpatient); 1 limited umbilical skin slough (conservative)	3 days (combined) vs 1 day ABP alone; 3 days TAH-BSO alone	Safe in appropriate patients; recent smokers and postbariatric patients are poor candidates; no PE in series
Kryger et al (2007)^8^	Literature review	N/A	Smoking cessation 3-6 weeks preoperative/postoperative; baseline hematocrit; VTE risk stratification; autologous donation if anticipated	N/A	Higher than individual procedures; check intraoperative hematocrit before ABP if substantial gynecologic blood loss	Separate instrument sets; normothermia; copious wound irrigation after gynecologic closure; ≥2 subcutaneous bulb-suction drains	PE remains greatest cause of mortality; obesity primary risk factor	Wound-healing complications higher in smokers; 85%-90% satisfaction	N/A	Safe in appropriate patients using multidisciplinary approach; PE is the greatest cause of mortality
Lai et al (2001)^10^	Case report	1	N/A	N/A	N/A	Cesarean section scar; endometrial cell implantation along incision route	Abdominal-wall endometrioma at scar	Cyclical painful abdominal-wall mass (menstruation-associated)	N/A	Abdominal-wall endometriosis is a recognized delayed complication; short-term follow-up will miss it
Massenburg et al (2015)^5^	Retrospective cohort	143	N/A	238 min (combined) vs 270 min (separate); *P* < .0001 Anesthesia time: 295 vs 364 min; *P* < .0001	Trend toward lower transfusion in combined group; *P* = .09 (NS)	N/A	Surgical complications 7.7% (combined) vs 12.5% (separate); *P* = .04 Pneumonia: 2.1% vs 0.5%; *P* = .04. PE 2.1% (NS). Trend to fewer reoperations (*P* = .08)	Superficial SSI 4.2%; seroma not captured in NSQIP	3.94 vs 4.44 days; *P* = .06 (trend)	Combined ABP–HYS has lower surgical complication rate than separate procedures; pneumonia and thromboprophylaxis essential
Matthes et al (1998)^11^	Case report	1	N/A	N/A	N/A	Combined ABP–HYS; extensive subcutaneous dissection creates potential space for endometrial implantation	Abdominal-wall endometrioma at lower abdominal scar—5 years postsurgery	Cyclical painful abdominal-wall mass (menstruation-associated)	N/A	Rare but recognized delayed complication; wide excision with clear margins recommended; short-term follow-up will miss it
Mou et al (2022)^6^	Retrospective cohort	269	N/A	244 min (combined) vs 168 min (ABP alone); ABP + MIH 38 min longer than ABP + AH (*P* = .008)	Transfusion 5.2% (combined) vs 3.4% (ABP alone); NS	Both AH and MIH routes included	No significant difference in 30-day complications (14.1% vs 11.5%; *P* = .22); HYS not associated with increased complications on multivariate analysis	Superficial SSI 4.1%; UTI 2.6%; transfusion 5.2%	2 days (combined) vs 1 day (ABP alone); ABP + MIH shorter than ABP + AH (1 vs 2 days; *P* < .001)	Combined ABP + HYS safe regardless of HYS route; smoking cessation essential
Perry (1986)^3^	Case series	20	N/A	N/A	1 transfusion required; no other significant blood loss	N/A	N/A	N/A	N/A	Safe in carefully selected patients; reduces convalescence and cost
Shull and Verheyden (1988)^12^	Case–control	33	Autotransfusion recommended; warmed blankets, IV fluids, anesthetic gases (normothermia); ABP markings preoperative	54% required >3 h; no significant per-procedure difference vs controls	Transfusion 21% (combined) vs 1.5% (controls); only 2 patients received >2 units	Sequential procedures; medically indicated procedure first; joint operative planning; meticulous hemostasis	1 PE; 2 reoperations (6%): 1 for breast bleeding, 1 for vaginal cuff bleeding	UTI 9%; other complications 9%; no wound infections in combined group	Combined group saved mean 1.92 days vs controls (*P* = .005)	No unusual hazards; complications are individual, not combination-related (except transfusion)
Simon et al (2006)^13^	Observational study	47	All patients treated as high DVT risk: heparin + pneumatic compression	N/A	No DVT/PE in combined ABP group; 1 saphenous vein thrombosis (morbidly obese panniculectomy + hernia patient, after flight)	N/A	No DVT or PE in combined ABP group	Overall 17% (combined) vs 18.5% (ABP alone); NS. Wound dehiscence 60% of complications; infection 20%; seroma 7%	N/A	Combining ABP with other procedures does not increase morbidity; obesity is the significant predictor of complications
Sinno et al (2011)^14^	Case–control	25	Sequential compression boots preinduction; prophylactic antibiotics 30 min preincision; incentive spirometer; early ambulation	221 min (combined) vs 277 min (sum of controls); *P* = .001	EBL 350 mL (combined) vs 551 mL (controls); *P* = .005; no transfusions	N/A	None (no transfusion, PE, reoperation, wound dehiscence, deep infection, or rehospitalization)	Wound infection 4%; seroma 4%	2.7 days (combined) vs 3.9 days (controls); *P* = .02	Combined surgery reduces OR time, blood loss, and hospital stay vs staged; no increase in complications
Voss et al (1986)^1^	Retrospective cohort	76	Prophylactic antibiotics; low-dose SC heparin; support stockings; incentive spirometry; prophylactic Coumadin	244 min (combined) vs 156 min (ABP alone); 19 min (gynecology alone)	EBL 551 mL; intraoperative transfusion 23.7% vs 7.9% controls; postoperative transfusion 35.5%	Extensive skin/subcutaneous undermining; SC drains in all patients; no suction lipectomy; rectus approximation in midline	5 PE (6.6%); no deaths; 2 reoperations (bowel obstruction; retained foreign body)	43.4% febrile morbidity; UTI 9.5%; wound infection 1.8%; cuff cellulitis 2.7%; atelectasis 24.8%	8.1 days (combined) vs 6.4 days (gynecology alone); 4.9 days (ABP alone)	Significantly increased morbidity; age >35 and weight >70 kg increase risk; anticoagulation warrants further evaluation

ABP, abdominoplasty; AH, abdominal hysterectomy; ASA, American Society of Anesthesiologists; BSO, bilateral salpingo-oophorectomy; COPD, chronic obstructive pulmonary disease; DVT, deep vein thrombosis; EBL, estimated blood loss; HYS, hysterectomy; MIH, minimally invasive hysterectomy; NGT, nasogastric tube; NS, not significant; NSQIP, National Surgical Quality Improvement Program; OR, operating room; PE, pulmonary embolism; SC, subcutaneous; SSI, surgical-site infection; TAH-BSO, total abdominal hysterectomy with bilateral salpingo-oophorectomy; UTI, urinary tract infection; VTE, venous thromboembolism; NA, not applicable; PDS, polydioxanone.

### Operative Time and Anesthesia

Operative time was reported in 7 studies. Voss et al recorded a mean combined operative time of 244 minutes vs 156 minutes for ABP alone; Kaplan and Bar-Meir reported 3.5 hours for the combined procedure vs 2.5 hours for ABP alone and 1.5 hours for total abdominal hysterectomy with BSO alone; and Gemperli et al and Gerow et al reported that the gynecologic component added ∼40 to 90 minutes. In contrast, Sinno et al reported a shorter combined operative time than the sum of staged controls (221 vs 277 minutes; *P* = .001), and Massenburg et al reported significantly shorter operative and anesthesia times in the combined group (238 vs 270 minutes and 295 vs 364 minutes, respectively; both *P* < .0001).

### Blood Loss and Transfusion

Voss et al reported intraoperative transfusion in 23.7% of combined patients vs 7.9% of controls, with a mean blood loss of 551 mL, and Shull and Verheyden reported a transfusion rate of 21% in combined patients vs 1.5% in controls. Sinno et al reported a lower estimated blood loss in the combined group than in staged controls (350 vs 551 mL; *P* = .005) with no transfusions required, and Massenburg et al reported a nonsignificant trend toward fewer transfusions in the combined group (*P* = .09).

### Complication Rates

Massenburg et al reported a lower overall surgical complication rate for combined ABP–hysterectomy than for staged procedures (7.7% vs 12.5%; *P* = .04), whereas Mou et al found no significant difference in 30-day complications by hysterectomy route (14.1% vs 11.5%; *P* = .22) and Simon et al reported no significant difference in overall complications (17% vs 18.5%). Superficial surgical-site infection (SSI) was reported at 4.2% by Massenburg et al and 4.1% by Mou et al. Simon et al reported wound dehiscence as the predominant complication (60% of all complications) with wound infection in 20%, and Kaplan and Bar-Meir reported 1 major wound infection requiring hospitalization. Massenburg et al reported a higher rate of pneumonia in the combined group (2.1% vs 0.5%; *P* = .04). Seroma was reported at ∼4% by Sinno et al.

### Venous Thromboembolism

Voss et al reported a pulmonary embolism (PE) rate of 6.6% in the combined group (5 events, no deaths), and Shull and Verheyden reported 1 PE. Simon et al recorded 1 superficial (saphenous) vein thrombosis and no deep venous thrombosis or PE. Studies that employed systematic VTE prophylaxis reported no deep venous thrombosis or PE events.

### Hospital Length of Stay

Massenburg et al reported a trend toward shorter length of stay in the combined group (3.94 vs 4.44 days; *P* = .06), Sinno et al reported a shorter stay for the combined group than the sum of staged controls (2.7 vs 3.9 days; *P* = .02), and Mou et al reported a shorter stay for combined ABP with minimally invasive hysterectomy than with abdominal hysterectomy (1 vs 2 days; *P* < .001). Earlier studies reported longer stays in the combined group, attributable in part to postoperative ileus.

### Delayed Complications

Two case reports described abdominal-wall endometrioma arising at the operative scar following combined ABP and hysterectomy (Matthes et al and Lai et al), presenting years after the index procedure.

## DISCUSSION

### Benefits of the Combined Approach

The concurrent performance of ABP and open gynecologic surgery offers several well-documented advantages. Combined procedures reduce total anesthesia exposure and shorten overall convalescence compared with 2 staged operations. It should be acknowledged, however, that ABP alone is frequently performed as an outpatient procedure in North America and that many gynecologic operations are likewise ambulatory; the reduction in hospitalization attributed to the combined approach is therefore not universal and depends on the specific gynecologic procedure and local practice, being most relevant where the indicated gynecologic operation already requires inpatient admission.^3,4,12^ Cost savings arise from a reduced requirement for duplicate perioperative resources, and the financial benefit is compounded by the fact that cosmetic procedures are not reimbursed by most insurance providers, making a single operative episode economically advantageous for the patient.^7,12^ From the gynecologic surgeon’s perspective, the combined approach affords improved exposure to pelvic anatomy.^5^ Patient satisfaction with the combined approach has been reported to exceed 85% to 90% across the available literature.^8^ Furthermore, a shorter single hospitalization has been associated with reduced exposure to nosocomial complications.^6^ Massenburg et al demonstrated that combined ABP–hysterectomy was associated with a significantly lower overall surgical complication rate compared with the 2 procedures performed separately (7.7% vs 12.5%; *P* = .04), along with shorter total operative and anesthesia times (*P* < .0001), supporting the efficiency and safety of the combined approach in appropriately selected patients.^5^

### Patient Selection and Risk Stratification

Careful patient selection is the cornerstone of safe combined surgery. Several modifiable and nonmodifiable risk factors have been consistently identified across the literature. Obesity is described as the single most important risk factor for postoperative complications, independently associated with VTE, increased intraoperative blood loss, wound-healing failure, and prolonged operative times.^1,8,13^ Voss et al reported that patients weighing >70 kg had significantly greater blood loss, and that age above 35 years was independently associated with increased risk.^1^ Accordingly, preoperative weight optimization through diet and exercise is strongly encouraged before elective combined procedures, given that ABP is a body contouring rather than a weight-reduction procedure.^6,8^

Smoking is a well-established modifiable risk factor that impedes wound healing through microvascular injury and is independently associated with postoperative wound complications following ABP, regardless of whether a concurrent gynecologic procedure is performed.^6,8^ Smoking cessation—including abstinence from nicotine replacement products such as patches and gum, given their vasoconstrictive effects—is recommended for a minimum of 3 to 6 weeks both before and after surgery.^8^ Patients who have recently ceased smoking and those with a history of bariatric surgery represent poor candidates for combined procedures, as demonstrated by Kaplan and Bar-Meir, whose only major wound infection occurred in such a patient.^7^ American Society of Anesthesiologists classification should be incorporated into preoperative risk stratification, and chronic obstructive pulmonary disease should be regarded as a relative contraindication to combined ABP, as fascial plication can reduce diaphragmatic excursion and precipitate respiratory compromise; this caution derives from limited, historical evidence (a single 1996 case series) and should be individualized according to disease severity and pulmonary reserve rather than applied as an absolute exclusion.^9^ The underlying gynecologic diagnosis should also inform candidacy. Suspected or known malignancy is a contraindication to concurrent ABP, as it may mandate staging, wider resection, or adjuvant therapy and introduces a risk of field contamination. Endometriosis warrants particular caution: extensive subcutaneous dissection in the presence of viable endometrial tissue may facilitate ectopic implantation along the incision, and abdominal-wall endometrioma is a recognized delayed complication of combined surgery; the potential for serious long-term morbidity means concurrent ABP should be approached conservatively, or deferred, in these patients. A history of bariatric surgery is best regarded as a relative rather than absolute contraindication: these patients frequently have residual micronutrient and protein deficiencies that impair wound healing, together with substantial residual skin laxity and altered abdominal-wall vascularity, all of which elevate the risk of wound-healing complications. Combined surgery may still be appropriate once weight has stabilized and nutritional status has been optimized and documented.

### Preoperative Planning

Effective preoperative planning requires close collaboration between the plastic and gynecologic surgical teams. Joint discussion of the operative plan, including incision placement, sequence of procedures, and anticipated blood loss, is essential before the procedure.^7,8^ Prophylactic antibiotics should be administered within 60 minutes before incision to minimize infectious morbidity. A first-generation cephalosporin (eg, cefazolin) is appropriate for most combined cases; weight-adjusted dosing should be used in obese patients, and intraoperative redosing is recommended for procedures exceeding the drug half-life (approximately every 3 to 4 hours for cefazolin) or where blood loss is substantial. Where vaginal entry or a higher risk of anaerobic contamination is anticipated, the gynecologic team's standard regimen (eg, the addition of metronidazole) should be followed.^1,8,14^ Venous thromboembolism risk stratification is mandatory and should employ a validated instrument, most commonly the 2005 Caprini Risk Assessment Model, which has been validated specifically in plastic and reconstructive surgery populations. In the 2005 Caprini framework, a score of 0 to 1 denotes low risk, 2 denotes moderate risk, 3 to 4 high risk, and 5 or greater the highest risk; combined ABP with an open gynecologic procedure typically accrues points for major surgery, prolonged operative time, malignancy or pelvic surgery, and frequently obesity or age, such that most candidates readily reach the high-risk category (Caprini 3-4) and frequently the highest-risk category (Caprini 5 or greater). Accordingly, several authors regard this combination as a routine indication for pharmacological prophylaxis. Postoperative low-molecular-weight heparin (LMWH; eg, enoxaparin) has been shown to reduce symptomatic VTE in high-risk plastic surgery patients and should be added to mechanical prophylaxis for patients at moderate or high risk, with timing balanced against bleeding risk.^1,2,7,15-18^ If substantial intraoperative blood loss is anticipated—for example, in patients undergoing hysterectomy for symptomatic fibroids—preoperative autologous blood donation should be considered.^8,12^ Baseline hematocrit measurement is also recommended to guide intraoperative decision making.^8^ Bowel preparation with preoperative bowel rest and intraoperative nasogastric tube decompression has been employed to reduce abdominal distension and facilitate wound closure.^9^ Nitrous oxide anesthesia is best avoided where feasible, as it may contribute to bowel distension; this recommendation rests on a single historical case report describing skin slough that required debridement and grafting and should therefore be regarded as low-grade evidence.^9^

### Intraoperative Considerations

The sequence of procedures should be determined preoperatively, with priority given to the medically indicated gynecologic procedure; this ensures that, should intraoperative complications necessitate early termination, the more essential component has been completed.^8,12^ Abdominoplasty skin markings should be performed by the plastic surgeon before anesthetic induction, before surgical positioning alters tissue relationships.^12^

Incision planning is a critical intraoperative consideration. A low transverse ABP incision suits both surgical teams and allows the gynecologic surgeon to plan the abdominal entry along or near the linea alba within the zone of planned excision, thereby minimizing scar burden.^6-8^ An inverted-T incision should be avoided in obese patients, as it carries a significantly higher risk of wound-healing complications.^8^ Gerow et al specifically advocate for French-line oblique incisions concealed within the bikini line, with the ABP skin-fat flap raised before peritoneal entry and gynecologic incisions kept at or near the midline.^9^

Meticulous hemostasis is essential, as blood loss is additive across both operative components.^12^ Intraoperative hematocrit should be measured following the gynecologic portion before proceeding with ABP, particularly when hemorrhage has been substantial.^8^ Separate instrument sets must be maintained between the gynecologic and plastic surgery components to reduce contamination risk and minimize infectious complications.^8^ Normothermia should be maintained throughout, as hypothermia is associated with increased SSI rates.^8,12^ Fascial plication should be performed in 2 layers using absorbable suture. Where the operating surgeon's routine ABP practice includes closed-suction drains, these are placed at the conclusion of the ABP component; drain use is a matter of surgeon preference and is not universal, and the addition of a gynecologic procedure does not in itself mandate drainage. Surgeons who ordinarily avoid drains—for example, using progressive-tension (quilting) sutures—may reasonably continue that practice, weighing the theoretical benefit of evacuating a potentially larger dead space against the morbidity associated with drains.^7,8^ The response to adverse intraoperative events should be agreed upon in advance. If excessive hemorrhage, hemodynamic instability, or gross contamination (eg, inadvertent bowel or bladder injury, or purulent or unexpected malignant findings) occurs during the gynecologic portion, the medically indicated procedure takes priority, and the elective ABP component should be curtailed or abandoned. In this circumstance, the team should proceed with simple wound closure rather than flap elevation and plication, thereby avoiding the additional operative time, dead space, and infection risk that ABP would introduce into an already compromised field.

### Operative Time

Operative duration in combined procedures is variable across the literature, and discrepancies between studies likely reflect differences in era, patient characteristics, and the extent of the combined procedures performed. Early studies reported significantly longer combined operative times: Voss et al recorded a mean of 244 minutes for combined ABP–hysterectomy compared with 156 minutes for ABP alone, whereas Gemperli et al and Gerow et al noted that the addition of the gynecologic component extended standard ABP time by ∼40 to 90 minutes.^1,2,9^ Kaplan and Bar-Meir reported a mean combined operative time of 3.5 hours, compared with 2.5 hours for ABP alone and 1.5 hours for TAH-BSO alone.^7^ In contrast, more recent comparative studies have demonstrated either equivalent or reduced total operative times for combined vs staged procedures: Sinno et al reported a significantly shorter operative time for combined surgery compared with the sum of individual controls (221 vs 277 minutes; *P* = .001), and Massenburg et al found significantly shorter operative and anesthesia times in the combined group (*P* < .0001).^5,14^ This trend likely reflects improved multidisciplinary coordination, refined patient selection, and advances in surgical technique over time. It should nonetheless be emphasized that the wider surgical literature consistently links prolonged operative duration to a higher incidence of complications, including venous thromboembolism, wound-healing problems, and infection; the more favorable operative times reported in recent combined series should therefore be interpreted with caution and do not negate the general principle that added operative time carries added risk. The evidence base is not uniformly concordant, and studies reporting longer combined operative times and greater morbidity must be weighed alongside the more recent comparative analyses rather than discounted.^19-21^

### Complication Profile and Prevention

#### Venous Thromboembolism

Venous thromboembolism remains the most serious reported complication of combined ABP and gynecologic surgery, and represents the leading cause of mortality in this patient population.^8^ Voss et al reported a PE rate of 6.6% in the combined group (5 events, no deaths), and Shull and Verheyden reported 1 PE in their series.^1,12^ Proposed mechanisms include elevated intra-abdominal pressure during surgery, pelvic venous compression, and postoperative immobilization.^1,8^ Notably, studies that implemented systematic VTE prophylaxis—including LMWH, sequential compression devices, and early ambulation—reported no deep venous thrombosis or PE events; the single thrombotic event recorded across these series was 1 superficial (saphenous) vein thrombosis reported by Simon et al, which did not constitute a deep venous or pulmonary thromboembolic event, underscoring the critical importance of prophylaxis.^2,7,13,14^ Sequential compression boots and incentive spirometry should be applied before induction and continued until mobilization, and LMWH should be prescribed for all moderate- to high-risk patients.^8^ Early ambulation is recommended for all patients regardless of VTE risk category.^14^ Because much of the historical guidance in this area predates current standards, contemporary VTE management should be anchored to present-day frameworks—formal Caprini scoring, risk-stratified chemoprophylaxis, and the aesthetic-surgery-specific guidance summarized by Pannucci—rather than to the 2007 review alone, and the perioperative algorithm (Figure 1) has been updated accordingly.^15-18^

**Figure 1.**
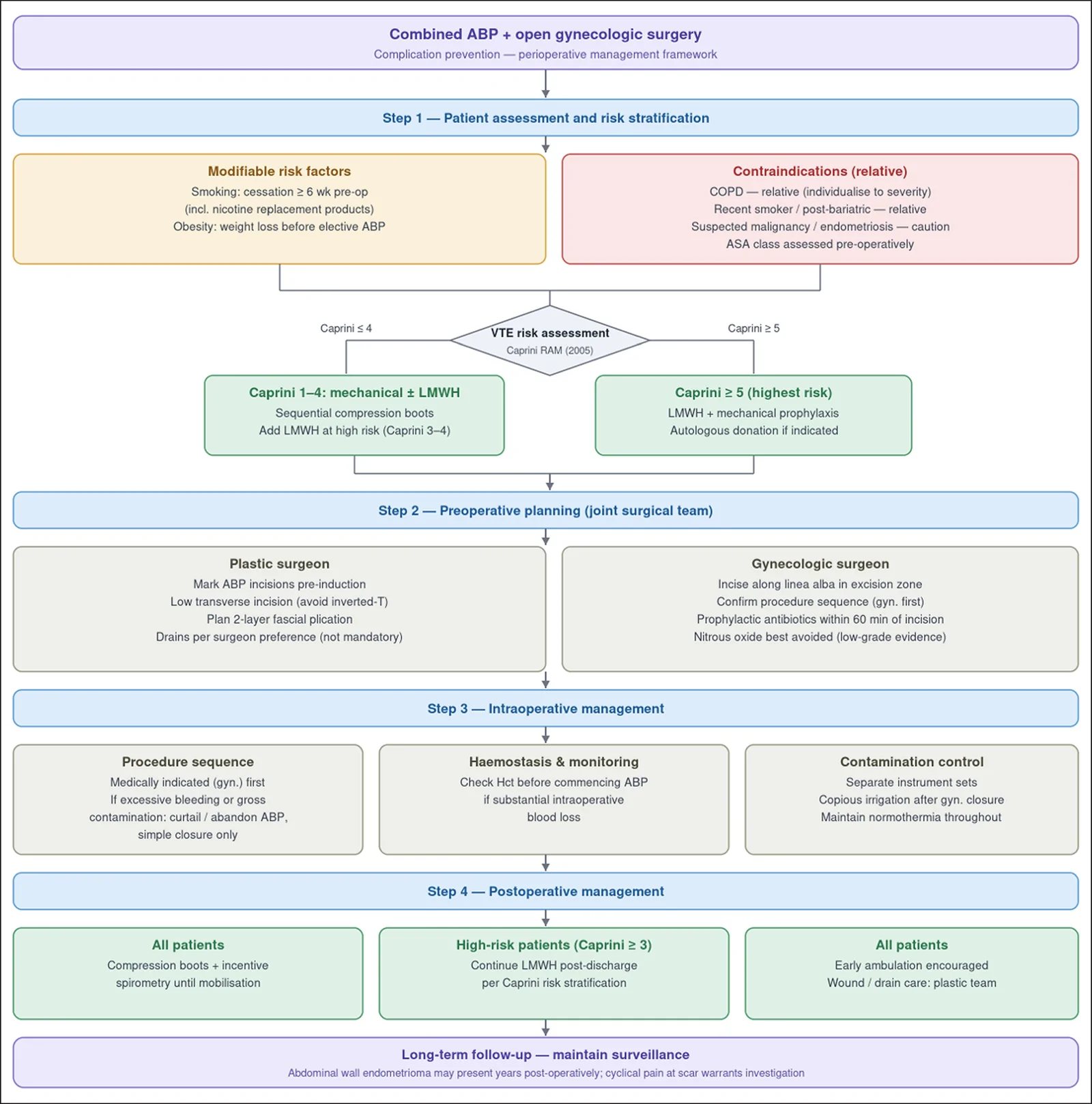
Multidisciplinary perioperative management algorithm for combined abdominoplasty and open gynecologic surgery. ABP, abdominoplasty; ASA, American Society of Anesthesiologists; COPD, chronic obstructive pulmonary disease; Hct, hematocrit; LMWH, low-molecular-weight heparin; VTE, venous thromboembolism.

#### Blood Loss and Transfusion

Intraoperative blood loss and transfusion requirements are consistently higher in combined procedures compared with ABP alone, reflecting the additive nature of hemorrhage across both operative components. Voss et al reported intraoperative transfusion in 23.7% of combined patients vs 7.9% of controls, with a mean blood loss of 551 mL, and Shull and Verheyden reported a significantly higher transfusion rate in combined patients (21% vs 1.5% in controls).^1,12^ In contrast, Sinno et al found significantly lower estimated blood loss in combined compared with staged procedures (350 vs 551 mL; *P* = .005) with no transfusions required, and Massenburg et al noted a trend toward fewer transfusions in the combined group (*P* = .09).^5,14^ These differences likely reflect improvements in patient selection and surgical technique over time. Autologous blood donation should be offered to patients in whom significant blood loss is anticipated, and intraoperative hematocrit monitoring is recommended before commencing the ABP component.^8,12^

#### Wound Complications and Infection

Wound-related morbidity is the most frequently reported minor complication category. Simon et al reported wound dehiscence as the predominant complication type (comprising 60% of all complications in their series), with wound infection in 20%.^13^ Mou et al reported a superficial SSI rate of 4.1%, comparable to the 4.2% reported by Massenburg et al.^5,6^ Kaplan and Bar-Meir reported 1 major wound infection requiring hospitalization and intravenous antibiotics, occurring in a recently quit smoker who had also undergone bariatric surgery, highlighting the importance of patient selection.^7^ Infectious morbidity appears to be significantly reduced by routine prophylactic antibiotics, maintenance of normothermia, the use of separate instrument sets for the 2 operative components, and copious wound irrigation following gynecologic closure.^1,8,14^

#### Pulmonary Complications

Beyond VTE, pneumonia represents an additional pulmonary risk associated with the combined approach. Massenburg et al reported a significantly higher rate of pneumonia in the combined ABP–hysterectomy group compared with separately staged procedures (2.1% vs 0.5%; *P* = .04), and recommended targeted pneumonia prophylaxis as part of perioperative management. The use of incentive spirometry in the postoperative period is consistently advocated.^5,8,14^

#### Seroma

Seroma formation is a recognized minor complication following ABP, with rates of ∼4% reported in combined procedure series.^14^ Gerow et al described seroma as a commonly encountered, outpatient-manageable annoyance.^9^ Subcutaneous drain placement is recommended routinely following the ABP component.^8^

#### Abdominal-Wall Endometrioma

A rare but clinically significant delayed complication of combined ABP and hysterectomy is abdominal-wall endometrioma. Two case reports in the included literature describe its occurrence: Matthes et al reported a patient who developed a cyclical painful mass at the operative scar 5 years after combined ABP–hysterectomy, and Lai et al described a similar endometrioma arising in a cesarean section scar.^10,11^ The proposed mechanism involves dissemination and implantation of endometrial cells along the incision route during extensive subcutaneous dissection, with the large potential space created during ABP facilitating implantation.^11^ Wide local excision with clear margins is the recommended treatment. Critically, this complication presents years after surgery and will not be captured by short-term follow-up studies, representing an important gap in the existing literature.

### Hospital Length of Stay

More recent studies consistently demonstrate that combined ABP–hysterectomy is associated with shorter or equivalent hospital stays compared with separately staged procedures. Massenburg et al reported a trend toward shorter length of stay in the combined group (3.94 vs 4.44 days; *P* = .06), and Mou et al demonstrated a significantly shorter stay following combined ABP with minimally invasive hysterectomy compared with combined ABP with abdominal hysterectomy (1 vs 2 days; *P* < .001).^5,6^ Sinno et al reported a significantly shorter hospital stay for the combined group compared with the sum of staged controls (2.7 vs 3.9 days; *P* = .02).^14^ In contrast, earlier studies reported longer stays in the combined group, attributable in part to postoperative ileus requiring monitoring.^1,9^ Discharge coordination is typically led by the gynecologic team, with both surgeons providing procedure-specific postoperative instructions to the patient.^8,12^ These comparisons should be interpreted in the context of local practice: where both component operations would ordinarily be performed on an ambulatory basis, the combined approach does not necessarily reduce, and may modestly increase, the likelihood of an overnight admission.

### Postoperative Management

Postoperative care should be coordinated between both surgical teams, with responsibilities allocated according to clinical domain. All patients should use sequential compression boots and incentive spirometry until discharge; continued LMWH thromboprophylaxis should be prescribed for high-risk patients.^8^ Wound care and, where drains have been placed, their management are directed by the plastic surgery team, whereas dietary and activity restrictions are governed by whichever operative component requires the more conservative recovery protocol.^8,12^ Early ambulation is encouraged universally.^14^ Both surgeons should independently counsel patients regarding their respective postoperative protocols to ensure compliance and appropriate follow-up.^12^ Long-term follow-up is particularly important given the risk of delayed complications such as abdominal-wall endometrioma, which may not manifest for several years postoperatively.^11^

Figure 1 illustrating a Multidisciplinary perioperative management algorithm for combined ABP and open gynecologic surgery.

### Limitations

Several limitations of the available evidence must be acknowledged. The majority of included studies employ retrospective designs—predominantly case series and case–control studies—with small sample sizes, limited statistical power, and the absence of randomized controls, all of which increase susceptibility to selection bias and residual confounding. Reliance on administrative databases such as the American College of Surgeons National Surgical Quality Improvement Program (ACS NSQIP) in 2 of the larger studies introduces the risk of coding inaccuracies and precludes capture of specific intraoperative variables, technique details, and long-term outcomes.^5,6^ Follow-up periods are predominantly limited to 30 days, which means that rare but clinically important delayed complications, such as abdominal-wall endometrioma, are systematically missed. Patient populations are heterogeneous with respect to BMI, comorbidity burden, and the specific gynecologic procedures combined with ABP, further limiting generalizability. The present review is itself subject to the inherent limitations of a nonsystematic narrative methodology, including potential selection bias in study inclusion and the inability to perform meta-analysis given outcome heterogeneity. Future research should prioritize prospective, multicenter studies with standardized outcome definitions, long-term follow-up, and sufficient sample sizes to enable robust subgroup analyses. Finally, by design this review addresses only combined procedures involving an open abdominal gynecologic component; as minimally invasive (laparoscopic and transvaginal) gynecologic surgery now predominates in many settings, the safety of combining ABP with these approaches, and any differences relative to open procedures, remains an important and largely unexplored question for future study.

## CONCLUSIONS

Combined ABP and open gynecologic surgery can be performed safely in appropriately selected patients, with complication rates that appear comparable to—and in some recent series lower than—those of staged procedures, although the evidence base is heterogeneous and not uniformly concordant and does not support presenting the combined approach as a definitive advantage for all patients. Safety is contingent upon rigorous patient selection, multidisciplinary preoperative planning, systematic VTE and infection prophylaxis, and coordinated postoperative management. The existing evidence base is limited by retrospective designs and outcome heterogeneity; prospective multicenter studies with standardized reporting and long-term follow-up are needed to establish definitive evidence-based criteria for this combined surgical approach.
